# Lipid Metabolic Reprogramming in Periodontitis-Associated Alveolar Bone Remodeling: Mechanisms, Signaling Pathways, and Therapeutic Implications

**DOI:** 10.3390/biom16091334

**Published:** 2026-09-14

**Authors:** Jinping Wang, Xiaorui Zhang, Yuxiao Zhang, Xiangyao Wang, Bowen Yang, Xiaojie Hu, Jing Mao, Zhixing Zhang, Haosen Li

**Affiliations:** 1 Department of Stomatology, Tongji Hospital, Tongji Medical College, Huazhong University of Science and Technology, Wuhan 430030, China; u202213415@hust.edu.cn (J.W.); u202211213@hust.edu.cn (X.Z.); m202372648@hust.edu.cn (Y.Z.); d202380929@hust.edu.cn (X.W.); u202413017@hust.edu.cn (B.Y.); m202672946@hust.edu.cn (X.H.); maojing@hust.edu.cn (J.M.); 2 School of Stomatology, Tongji Medical College, Huazhong University of Science and Technology, Wuhan 430030, China; 3 Hubei Province Key Laboratory of Oral and Maxillofacial Development and Regeneration, Wuhan 430030, China

**Keywords:** lipid metabolic reprogramming, periodontitis, alveolar bone remodeling, osteoimmunology, oxidative stress, periodontal regeneration

## Abstract

Periodontitis-associated alveolar bone loss reflects not only persistent inflammation but also profound metabolic remodeling within the periodontal microenvironment. This review synthesizes current evidence on how lipid metabolic reprogramming regulates periodontal bone remodeling and its therapeutic significance. Under physiological conditions, fatty acid uptake and β-oxidation, lipid droplet turnover, and lipid-derived signaling support osteoblast bioenergetics, stem-cell osteogenesis, and balanced bone remodeling. In contrast, dyslipidemia and chronic inflammatory stress promote the accumulation of free fatty acids, oxidized lipoproteins, cholesterol derivatives, and sphingolipid metabolites, thereby enhancing reactive oxygen species production, lipid peroxidation, inflammasome activation, and pro-inflammatory immune polarization. These changes suppress osteogenic differentiation, stimulate osteoclastogenesis, and impair periodontal regeneration. We further integrate major regulatory networks, including PPARγ–Wnt/β-catenin, AMPK–mTOR, NF-κB/NLRP3, and GSK3β–NRF2/ferroptosis signaling. Therapeutic strategies targeting these lipid–redox–immune circuits include specialized pro-resolving lipid mediators, n-3 polyunsaturated fatty acids, statins, metabolic modulators, and local biomaterial- or nanocarrier-based delivery systems. Accordingly, lipid metabolic reprogramming may provide a framework for developing metabolism-oriented adjunctive and regenerative strategies for periodontitis.

## 1. Introduction

Periodontitis is a biofilm-associated, host-mediated chronic inflammatory disease characterized by the progressive destruction of the tooth-supporting apparatus, including the periodontal ligament, cementum, and alveolar bone [1,2]. In this review, periodontitis refers specifically to marginal periodontitis driven by dysbiotic subgingival biofilms and a dysregulated host response, rather than apical periodontitis of endodontic origin. Its most consequential pathological outcome is the disruption of physiological bone remodeling, in which excessive osteoclast-mediated resorption is insufficiently counterbalanced by osteoblast-mediated bone formation and regenerative activity of periodontal progenitor cells [3]. This imbalance results in progressive alveolar bone loss that is often difficult to reverse clinically. Although dysbiotic microbial communities initiate the disease, the magnitude and persistence of tissue destruction are determined by the host response and by the local microenvironment in which immune, stromal, and bone-lineage cells interact [2,3].

Cellular metabolism provides more than energy for periodontal tissues. Dynamic changes in glycolysis, mitochondrial oxidative phosphorylation, amino-acid utilization, and lipid metabolism influence the availability of ATP and biosynthetic precursors, redox homeostasis, epigenetic regulation, and inflammatory effector functions [4]. Among these processes, lipid metabolism is particularly relevant because fatty acids, cholesterol, phospholipids, and sphingolipids are not only structural or energy-storage molecules but also precursors of signaling mediators that regulate immune activation, inflammation resolution, and cell-fate decisions [4,5]. Under physiological conditions, coordinated lipid uptake, storage, mobilization, and oxidation support cellular homeostasis and tissue repair. In contrast, excessive fatty-acid exposure, disturbed cholesterol handling, oxidized-lipid accumulation, and altered lipid-mediator production may promote lipotoxic stress, persistent inflammation, and impaired regeneration [4,5,6].

This distinction is especially important in periodontitis. Normal inflammation is transient and is followed by active resolution, during which pro-resolving lipid mediators help restrain excessive leukocyte activation, promote clearance of inflammatory debris, and restore tissue homeostasis [7]. In periodontitis, however, persistent dysbiosis and inflammatory stimulation occur within a microenvironment that may be further shaped by systemic metabolic comorbidities, including dyslipidemia, obesity, and diabetes [2,8]. Recent clinical evidence supports an association between periodontitis and unfavorable circulating lipid profiles, although these epidemiological observations should not be interpreted as proof of local causality [8]. Mechanistic studies have begun to provide more direct evidence: cholesterol overload can disrupt cholesterol homeostasis in gingival fibroblasts, induce inflammatory signaling, and aggravate alveolar bone loss in experimental models [9]. Likewise, impaired lipid-related signaling in periodontal cells can compromise osteogenic capacity and intensify inflammation and bone loss [10]. Together, these findings support the concept that abnormal lipid metabolism may act not only as a systemic comorbidity marker but also as a local modifier of periodontal inflammatory and bone-remodeling responses.

In this review, lipid metabolic reprogramming refers specifically to adaptive or maladaptive changes in lipid uptake, de novo synthesis, storage in lipid droplets, lipolysis, fatty-acid oxidation, cholesterol trafficking, and the production of lipid-derived mediators in response to inflammatory, microbial, or metabolic stress. This definition distinguishes metabolic reprogramming from a simple increase in circulating lipid concentrations. Through these processes, lipid perturbations may reshape the functions of osteoblast-lineage cells, osteoclasts, periodontal ligament stem cells, fibroblasts, and immune cells, thereby influencing the coupling of bone formation and resorption.

Several recent reviews have advanced the field by examining the bidirectional association between periodontitis and dyslipidemia [8], lipid metabolism and lipid-lowering drugs in periodontal disease [11], diabetes-associated lipotoxicity [6], and macrophage metabolic reprogramming [12]. However, these perspectives do not fully center periodontal bone remodeling as the principal biological and clinical endpoint, nor do they consistently integrate lipid metabolic alterations across bone-lineage, stromal, and immune-cell populations. In addition, evidence remains uneven across study systems: causal mechanisms are largely supported by in vitro experiments and animal models, whereas human evidence is dominated by cross-sectional associations, circulating lipid measurements, and limited lipidomic datasets [8,13]. A critical synthesis is therefore needed to distinguish direct mechanistic evidence from association, identify convergent versus cell-specific pathways, and clarify the translational distance between metabolic targets and clinically meaningful periodontal regeneration.

Accordingly, this review examines lipid metabolic reprogramming as a mechanistic interface linking dysbiotic inflammation to periodontal bone remodeling. We integrate evidence on how altered lipid flux and lipid-derived signals regulate osteoblasts, osteoclasts, periodontal ligament stem cells, fibroblasts, and immune cells; analyze the crosstalk among lipid, redox, inflammatory, and osteogenic pathways; and assess therapeutic opportunities aimed at restoring metabolic homeostasis. Throughout, we distinguish findings derived from cell culture, animal models, and human studies, with particular attention to unresolved causal, temporal, and translational questions. By placing periodontal bone remodeling at the center of the analysis, this review aims to provide a more integrated framework for metabolism-oriented prevention and regenerative therapy in periodontitis.

## 2. Multifaceted Regulatory Roles of Lipid Metabolism in Periodontal Bone Remodeling

Lipid metabolism supports periodontal bone remodeling by providing energy, guiding cell differentiation, and coordinating bone–immune interactions. These functions help maintain the balance between bone formation and resorption.

Fatty acid oxidation is an important energy source for osteoblast differentiation and mineralization. Lipid droplets store fatty acids as intracellular energy reserves [14], which are released by ATGL-mediated lipolysis and oxidized in mitochondria to generate ATP for bone matrix synthesis [15]. Pharmacological inhibition of ATGL reduces ATP production, and exogenous glucose does not restore this deficit, indicating that osteoblasts cannot readily replace fatty acid oxidation with glucose metabolism [15]. LRP5 stabilizes β-catenin and induces c-FOS and enzymes involved in fatty acid oxidation, whereas *Cpt2* facilitates mitochondrial oxidation. Loss of LRP5 signaling or *Cpt2* reduces ATP generation and impairs mineralization [16,17]. Together, these findings show that fatty acid oxidation provides the energy required for normal osteoblast function.

Fatty acid composition also influences the balance between bone formation and resorption. n-6 polyunsaturated fatty acids, such as arachidonic acid, can favor adipogenic differentiation and promote osteoclastogenesis through PPARγ and PGE_2_–RANKL signaling. By contrast, n-3 polyunsaturated fatty acids, including eicosapentaenoic acid and docosahexaenoic acid, support osteogenic signaling and limit osteoclast formation [18,19,20,21]. Although this evidence comes from different cell and animal models, it consistently suggests that lipid quality, rather than lipid abundance alone, influences bone-cell behavior.

Immune cells provide another link between lipid metabolism and bone remodeling. In skeletal repair models, fatty acids released from apoptotic chondrocytes are taken up by macrophages through macrophage scavenger receptor 1 and used for fatty acid oxidation. The resulting increase in NAD^+^ activates the SIRT1/EZH2 axis and promotes BMP2 secretion, creating a pro-osteogenic environment [22]. Although this evidence is not periodontal-specific, it shows how lipid utilization by macrophages can support bone repair.

Cholesterol and adipokines further influence the osteoblast–osteoclast balance. Excess cholesterol can promote osteoclastogenesis by increasing the RANKL/OPG ratio and supporting Hedgehog signaling [23,24,25]. Adipokines have fewer uniform effects: chemerin tends to promote bone resorption [26,27], whereas visfatin and omentin-1 may suppress osteoclast-related markers or increase the OPG/RANKL ratio [28,29,30,31]. These differences show that adipokine effects depend on the specific mediator and cellular context.

Sphingolipid metabolites also have distinct effects on bone cells. Balanced sphingomyelin metabolism supports mineralization, whereas ceramide reduces osteoblast viability and is associated with bone resorption. In contrast, sphingosine-1-phosphate promotes osteoblast survival and bone formation through S1P receptors [32]. Thus, the balance among individual sphingolipid metabolites is more informative than their total abundance.

**Figure 1:**
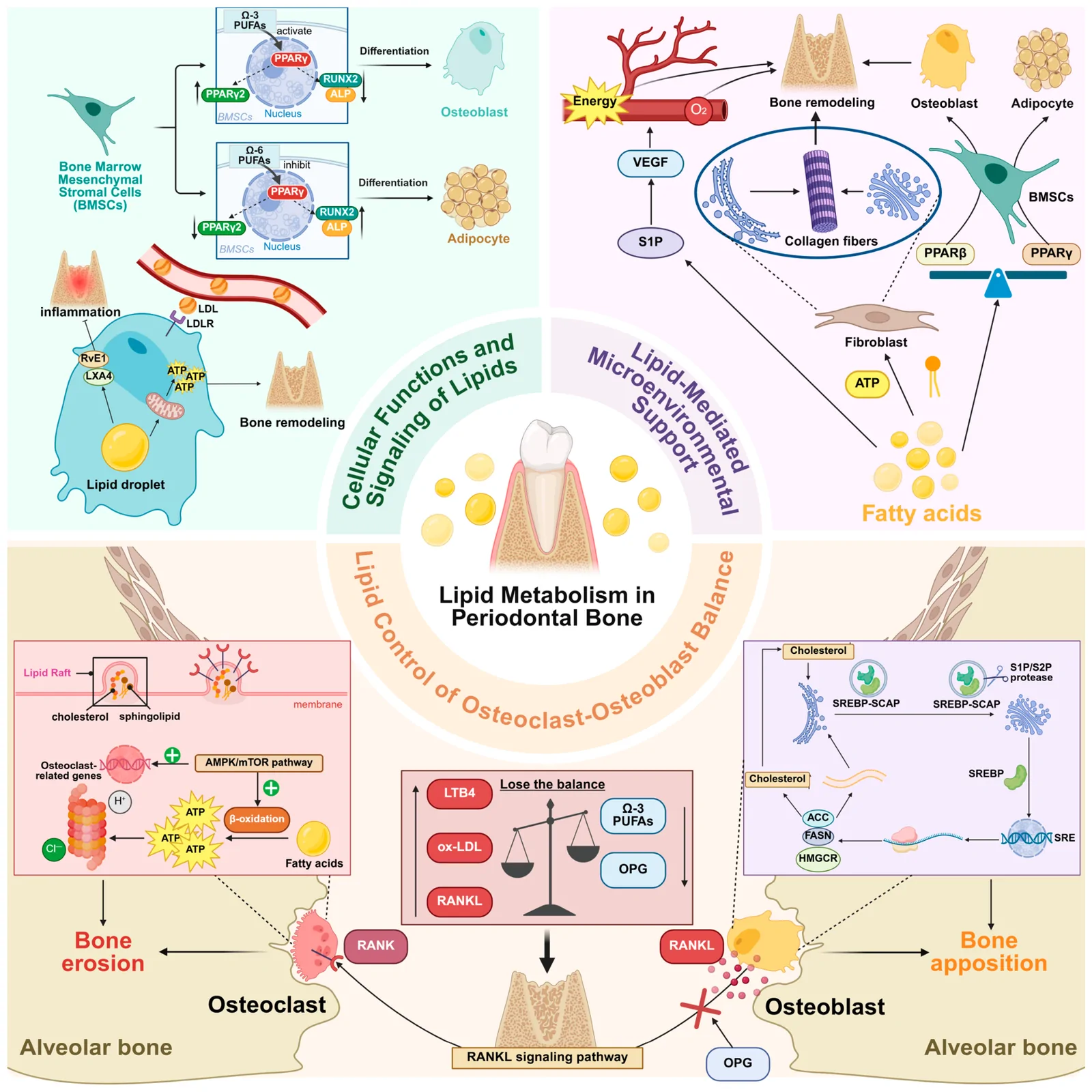
Lipid metabolism’s role in periodontal bone homeostasis. Lipids provide energy, structural components, and signaling molecules that regulate cellular processes, tissue microenvironment, and osteoclast-osteoblast balance. In periodontitis, disrupted lipid metabolism shifts this balance towards pro-osteoclastogenic signaling, leading to net bone loss. Created in BioRender. Yuxiao, Z. (2026) https://BioRender.com/yseg1aj (accessed on 31 August 2026).

Overall, lipid metabolism supports periodontal bone homeostasis by supplying energy, shaping bone-cell differentiation, and coordinating bone–immune signaling. Its effects vary with lipid type and biological context. When this balance is disrupted, these normal regulatory functions shift toward inflammation, impaired regeneration, and bone loss, as discussed in Section 3. The principal functions of lipid metabolism in maintaining periodontal bone homeostasis are summarized in Figure 1.

## 3. Molecular Mechanisms of Lipid Metabolism and Periodontal Bone Remodeling

### 3.1. Inflammatory and Oxidative Mechanisms of Lipid Metabolism Disorders in the Periodontal Bone Microenvironment

As discussed in Section 2, physiological lipid metabolism does not itself damage alveolar bone. Lipid mediators normally coordinate the initiation and timely resolution of inflammation, thereby maintaining periodontal homeostasis [33]. In periodontitis, persistent pro-inflammatory lipid signaling and insufficient or delayed resolution convert a self-limiting host defense into sustained inflammation and tissue damage, ultimately uncoupling alveolar bone resorption from formation [34].

Lipid dysregulation reshapes the periodontal microenvironment by increasing the fatty acid burden, promoting oxidative lipid modification, and disrupting the lipid mediator profile [13]. In this altered milieu, experimental studies suggest that saturated fatty acids (SFAs) amplify inflammatory signaling in periodontal cells [35]. Oxidized low-density lipoprotein (ox-LDL), however, acts in a cell-specific manner: it promotes osteoclastogenesis in Toll-like receptor 2 (TLR2)-primed bone marrow precursor cells via lectin-like oxidized low-density lipoprotein receptor-1 (LOX-1), but minimally affects the differentiation of human gingival mesenchymal stem cells despite reducing their proliferation [36,37]. Arachidonic acid-derived PGE_2_ and leukotriene B_4_ (LTB_4_) contribute to inflammatory initiation and amplification, whereas EPA- and DHA-derived mediators, such as resolvins, limit continued neutrophil recruitment and promote inflammation resolution [38]. Overall, lipid dysregulation may sustain periodontal inflammation by strengthening pro-inflammatory and oxidized lipid signaling while weakening pro-resolving responses, thereby favoring bone resorption and exacerbating alveolar bone loss.

In experimental periodontal and non-periodontal systems, microbial and metabolic stressors have been linked to overlapping NF-κB-, ROS-, NLRP3-, and ferroptosis-related processes [39,40,41,42,43]. These processes may jointly impair bone formation and enhance osteoclastogenic signaling, although no single periodontal study has established the entire sequence as one continuous cascade.

**Figure 2:**
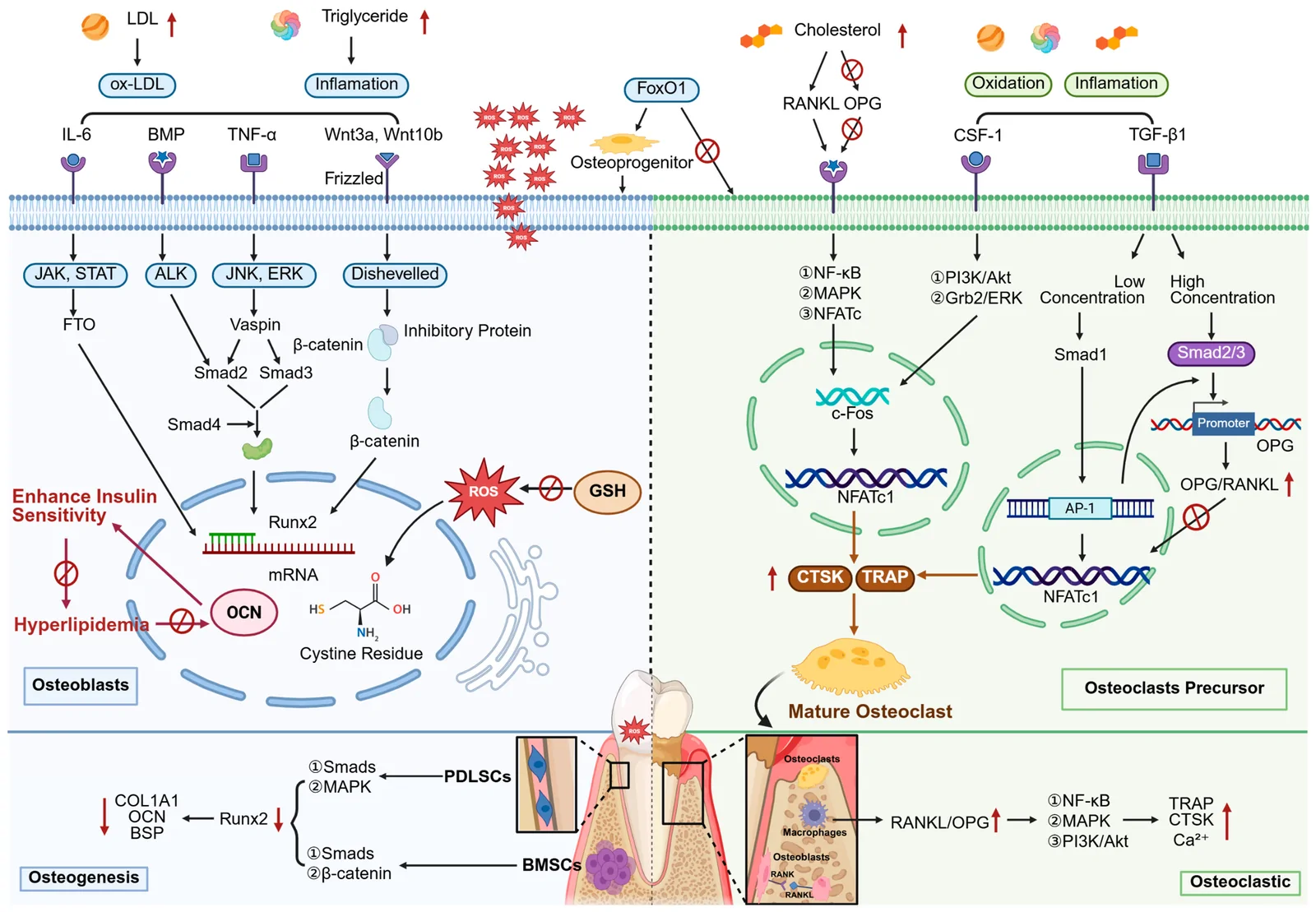
Lipid metabolism disorders disrupt periodontal bone homeostasis. They suppress osteogenesis by inhibiting Wnt/β-catenin/Smad pathways and reducing Runx2 expression in osteoblasts. Concurrently, they promote osteoclastogenesis via PI3K/Akt, NF-κB/MAPK, and TGF-β/Smad pathways, enhancing NFATc1-mediated maturation. This imbalance in bone remodeling leads to periodontal bone destruction. Created in BioRender. Yuxiao, Z. (2026) https://BioRender.com/n1eyj6d (accessed on 31 August 2026).

However, direct evidence linking lipid dysregulation to periodontal bone remodeling remains limited. Human studies mainly show correlations, whereas high-fat diet models are influenced by concurrent changes in body weight, insulin sensitivity, and the microbiota. Cell studies reveal possible mechanisms, but the lipid doses used may not reflect actual periodontal exposure, and findings from non-periodontal models provide only indirect support. Thus, lipid dysregulation is a plausible contributor to alveolar bone loss, but its causal role in humans remains unconfirmed. The principal local mechanisms are summarized in Figure 2.

### 3.2. Functional Adaptation and Differentiation Balance of Multiple Stem Cells Under Lipid Metabolic Stress

Periodontal and dental stem cells differ in their relevance to lipid-associated periodontal damage according to their location, regenerative role, and evidence strength.

Among the stem-cell populations reviewed, PDLSCs have the strongest direct evidence because their location at the periodontal ligament–alveolar bone interface makes them central to periodontal maintenance and regeneration. In vitro, palmitate reduced PDLSC proliferation, osteogenic differentiation, and mineralization while increasing apoptosis and inflammatory cytokine expression [44]. Another study confirmed the impaired osteogenesis and showed that palmitate downregulated lncRNA *AC018926.2*, thereby suppressing ITGA2/FAK/AKT signaling [45]. Together, these findings provide consistent evidence of palmitate-induced PDLSC dysfunction, although their clinical relevance remains uncertain because both studies were conducted in vitro. Moreover, lipid-related signals do not affect PDLSCs uniformly. The bacterial short-chain fatty acid butyrate inhibited osteogenesis through FFAR2–Smad1 signaling [46], whereas cholesterol-derived oxysterols activated LXR–Hedgehog signaling and promoted PDLSC osteogenesis and alveolar bone healing [47]. These contrasting effects indicate that PDLSC function depends on the type and source of lipid signals rather than lipid exposure alone.

Gingiva-derived mesenchymal stem cells (GMSCs) and BMSCs provide complementary but less direct evidence. Transplantation of human GMSCs into hyperlipidemic mice with periodontitis reduced circulating lipids, inflammation, and alveolar bone loss while promoting new bone formation [48]. BMSCs contribute more directly to bone formation in periodontal tissues. In obese mice with periodontitis, impaired AMPK-dependent fatty-acid oxidation suppressed BMSC osteogenesis, whereas metabolic restoration promoted alveolar bone regeneration [49]. Thus, GMSCs appear more relevant to immunomodulation, whereas BMSCs primarily support osseous repair; evidence for both remains preclinical. Direct comparisons further clarify differences in regenerative potential. Under identical culture conditions, PDLSCs showed the greatest osteogenic capacity, followed by supracrestal gingiva-derived mesenchymal stem cells (SG-GMSCs) and marginal gingiva-derived mesenchymal stem cells (MG-GMSCs); adipogenic capacity was similar across the three populations [50]. A same-donor comparison of DPSCs, PDLSCs, DFPCs, and alveolar bone marrow mesenchymal stem cells (ABMMSCs) found that ABMMSCs and PDLSCs had the highest osteogenic capacity, followed by DPSCs, whereas DFPCs showed the lowest capacity [51]. Thus, these studies show which cell types have greater regenerative potential under standard culture conditions, but not which are more resistant to lipid stress.

Evidence for dental follicle cells (DFCs) and dental pulp stem cells (DPSCs) is more preliminary. Osteogenic differentiation of DFCs was accompanied by lipidomic remodeling and required fatty-acid synthesis [52], whereas cholesterol-rich lipid rafts regulated AKT-dependent differentiation in DPSCs [53].

Taken together, PDLSCs provide the clearest evidence that lipid signals can alter periodontal regeneration, whereas evidence for other stem-cell populations remains mainly preclinical or mechanistic. Standardized comparisons and in vivo validation are now needed to determine which populations best retain regenerative function under pathological lipid stress.

### 3.3. Integrated Signaling Mechanisms Linking Lipid Metabolism to Periodontal Bone Remodeling

Direct lipid perturbation has been examined most clearly for the PPARγ–Wnt and AMPK–mTOR axes, whereas the links between lipid stress and Notch–BMP signaling in periodontal tissues remain comparatively indirect. Peroxisome proliferator-activated receptor-γ (PPARγ) and Wnt/β-catenin regulate osteogenic–adipogenic differentiation, whereas the AMP-activated protein kinase (AMPK)–mechanistic target of rapamycin (mTOR) axis coordinates energy metabolism and autophagy. Notch, leptin, and bone morphogenetic protein (BMP) signaling further shape bone formation and osteoclast-mediated resorption. Together, this network determines whether periodontal tissues regenerate or undergo bone loss under lipid metabolic stress.

The PPARγ–Wnt axis is a key regulator of the balance between osteogenic and adipogenic differentiation. However, the role of PPARγ in periodontal repair is highly context-dependent rather than uniformly detrimental. In human periodontal ligament fibroblasts, PPARγ supported osteogenic and cementogenic differentiation by maintaining H3K27 acetylation and the expression of lineage-related genes [54]. By contrast, in PDLSCs, platelet-derived growth factor-BB promoted osteogenesis and suppressed adipogenic differentiation by increasing *Wnt1*, β-catenin, and *BMP2* expression while decreasing *PPARγ* and *GSK3β* expression [55]. Overall, PPARγ can either promote or inhibit osteogenic differentiation, depending on the cell state and upstream signals. A similarly stage-dependent effect has been observed in vivo. In experimental periodontitis, early PPARγ activation alleviated inflammation and bone loss. However, prolonged activation reduced OPG, *RUNX2*, and *BMP2* expression, increased RANKL expression, and ultimately exacerbated bone loss [56]. Therefore, the short-term anti-inflammatory effects of PPARγ activation should not be assumed to confer long-term regenerative benefits.

Wnt/β-catenin is a major pathway that promotes osteogenic differentiation and is regulated by both inflammatory and metabolic factors. In PDLSCs, lipopolysaccharide reduced lysine lactylation, inhibited Wnt/β-catenin signaling, and impaired osteogenesis, whereas proanthocyanidins reversed these effects [57]. Similarly, β-catenin activation promoted cellular cementum formation in vivo [58]. However, the final cellular response also depends on AMPK–mTOR signaling. Under short-term nutrient deprivation, human periodontal ligament cells activated ROS–AMPK–mTOR signaling and autophagy to adapt to stress. When nutrient deprivation was prolonged, this protective response declined and cell death increased [59]. High extracellular lactate also activated MCT1–mTOR signaling, suppressed autophagy, and inhibited osteogenic differentiation of PDLSCs; these effects were partly reversed by rapamycin [60]. This finding does not contradict the positive relationship between intracellular lactylation and Wnt activity [57], because extracellular lactate signaling and intracellular protein lactylation are distinct processes. Consistent with the protective role of autophagy, metformin suppressed mTOR signaling, restored autophagy disrupted by lipopolysaccharide, reduced HMGB1-associated oxidative stress, and limited alveolar bone loss in experimental periodontitis [61]. Thus, AMPK–mTOR signaling regulates osteogenic differentiation by controlling how periodontal cells respond to metabolic stress.

The effects of Notch and BMP signaling also depend on the biological context. In human periodontal ligament cells, Jagged1–NOTCH2 signaling increased osteogenic gene expression and mineralization [62]. However, it also reduced OPG expression without affecting RANKL, causing the conditioned medium to promote osteoclast formation [63]. These findings are not necessarily contradictory: Jagged1–NOTCH2 may directly promote osteogenic differentiation while indirectly stimulating osteoclast formation. Studies in human mesenchymal stem cells have also shown that canonical Notch signaling is required for BMP2- and BMP6-induced osteoblast differentiation, although this interaction has not yet been confirmed in periodontal tissues [64]. In inflamed PDLSCs, BMP9 promoted osteogenesis but had little effect on inflammation. The addition of NELL1 further enhanced BMP9-induced bone formation and reduced inflammatory gene expression through p38/ERK signaling [65]. Thus, the effects of BMP signaling depend on its interaction with Notch signaling and the surrounding inflammatory conditions.

Leptin adds a systemic metabolic input to this local network. In human gingival tissues, leptin correlated positively with OPG and negatively with RANKL. In cultured gingival fibroblasts, low leptin increased the OPG/RANKL ratio, whereas high leptin produced the opposite effect [66], showing that its action is dose-dependent. Leptin-receptor expression should not, however, be treated as direct evidence of leptin signaling. In LepR-positive alveolar stromal cells, periodontitis induced CCRL2, which bound SFRP1 and strengthened Wnt inhibition, thereby impairing socket bone healing [67]. This study identifies inflammation–Wnt crosstalk in a leptin-receptor-positive population, but does not prove that leptin itself caused the defect.

Overall, these pathways act as a connected network. PPARγ and Wnt/β-catenin regulate cell differentiation, AMPK–mTOR controls energy metabolism and autophagy, and other signals further influence bone formation and resorption. Differences among studies may reflect cell type, signal intensity, and exposure time. Because direct periodontal evidence remains limited, future studies should examine these pathways together under clinically relevant lipid conditions. The major lipid-sensitive pathways, their principal targets, and their implications for periodontal bone remodeling are summarized in Table 1.

## 4. Disruptive Effects of Abnormal Lipid Metabolism on Periodontal Bone Remodeling

Section 3 addressed how lipid-sensitive pathways intersect mechanistically. This section considers the same evidence from an outcome-oriented perspective by comparing cell-level regenerative failure, tissue-level inflammatory destruction, and systemic metabolic associations, while distinguishing cell, animal, and human evidence.

### 4.1. Cell-Level Loss of Periodontal Regenerative Competence Under Lipid Overload

At the cellular level, lipid overload reduces the regenerative reserve of periodontal tissues. As detailed in Section 3.2, palmitate compromises PDLSC survival and osteogenic capacity [44,45]. These periodontal-specific findings are derived from in vitro experiments; they therefore demonstrate cellular susceptibility to lipotoxic stress but do not establish clinical causality.

Recent work further links hyperlipidemia to regenerative failure through redox-sensitive cell death. In a high-fat-diet-associated periodontitis model, dysregulated lipid metabolism induced mitochondrial dysfunction, lipid peroxidation, and ferroptosis in PDLSCs, with reduced osteogenic differentiation and aggravated alveolar bone loss; local inhibition of ferroptosis or GSK3β partly restored osteogenic and periodontal bone parameters [42]. Because this study combined cell experiments with a mouse model, it provides stronger preclinical support than isolated in vitro observations. Nevertheless, high-fat feeding cannot separate lipid-specific effects from concurrent obesity, insulin resistance, or microbiome alterations.

Cholesterol dysregulation identifies gingival fibroblasts as a second local cellular target. Disturbed cholesterol homeostasis increased inflammatory mediator production in human gingival fibroblasts and aggravated periodontal inflammation and alveolar bone loss in experimental models [9]. More recent work combining human periodontal single-cell data with rat and cell experiments identified gingival fibroblasts as strongly cholesterol-responsive and linked cholesterol overload to lysosomal injury, cathepsin-B-dependent NLRP3 activation, pyroptosis, and alveolar bone loss [68]. Because functional testing remained preclinical, the same causal sequence has not yet been demonstrated in patients.

Taken together, the available evidence supports a tiered interpretation: human periodontal cell studies consistently indicate loss of regenerative or homeostatic function, and animal models show compatible tissue destruction, but direct human evidence that lipid overload independently causes periodontal regenerative failure remains lacking.

### 4.2. Tissue-Level Lipid–Redox–Immune Amplification and Impaired Resolution

Beyond the cell-level injury described in Section 4.1, abnormal lipid metabolism may amplify periodontal destruction at the tissue level. High-fat-diet-induced metabolic syndrome aggravated ligature-induced alveolar bone loss, while complementary palmitate/LPS experiments demonstrated enhanced macrophage osteoclast formation and inflammatory gene expression [43]. As in the model discussed in Section 4.1, these findings support an integrated metabolic susceptibility rather than a lipid-only causal effect.

Human gingival lipidomics provides direct evidence that the local lipid environment is altered in periodontitis. Gingival tissues from patients with periodontitis showed increased lysophosphatidylcholines, diacylglycerols, and phosphatidylethanolamines, together with reduced triacylglycerols and increased expression of lipid-handling enzymes, including secretory phospholipase A2 [69]. This pattern is consistent with altered membrane-phospholipid turnover and greater availability of lipid-derived signaling substrates in inflamed tissues. Oxidative stress may reinforce this remodeling by promoting lipid peroxidation and extracellular-matrix injury. Consistent with this interpretation, a systematic review and meta-analysis reported higher levels of malondialdehyde in patients with chronic periodontitis [70]. These human data demonstrate an association with a remodeled lipid–redox environment, but do not establish whether the lipid changes initiate or result from periodontal inflammation.

The more consistent mechanistic implication is that altered lipid handling may impair inflammatory resolution rather than simply increase pro-inflammatory signaling. In a human integrated analysis, gingival lipid-mediator profiles and specialized pro-resolving mediator (SPM) receptor expression differed among periodontal health, untreated periodontitis, and post-treatment periodontitis and were associated with subgingival microbial profiles [7]. Lower salivary lipoxin A4 levels were also associated with worse periodontal clinical parameters [71]. These findings support dysregulation of resolution pathways, although they do not imply that all SPMs are uniformly reduced across patients or disease stages.

**Figure 3:**
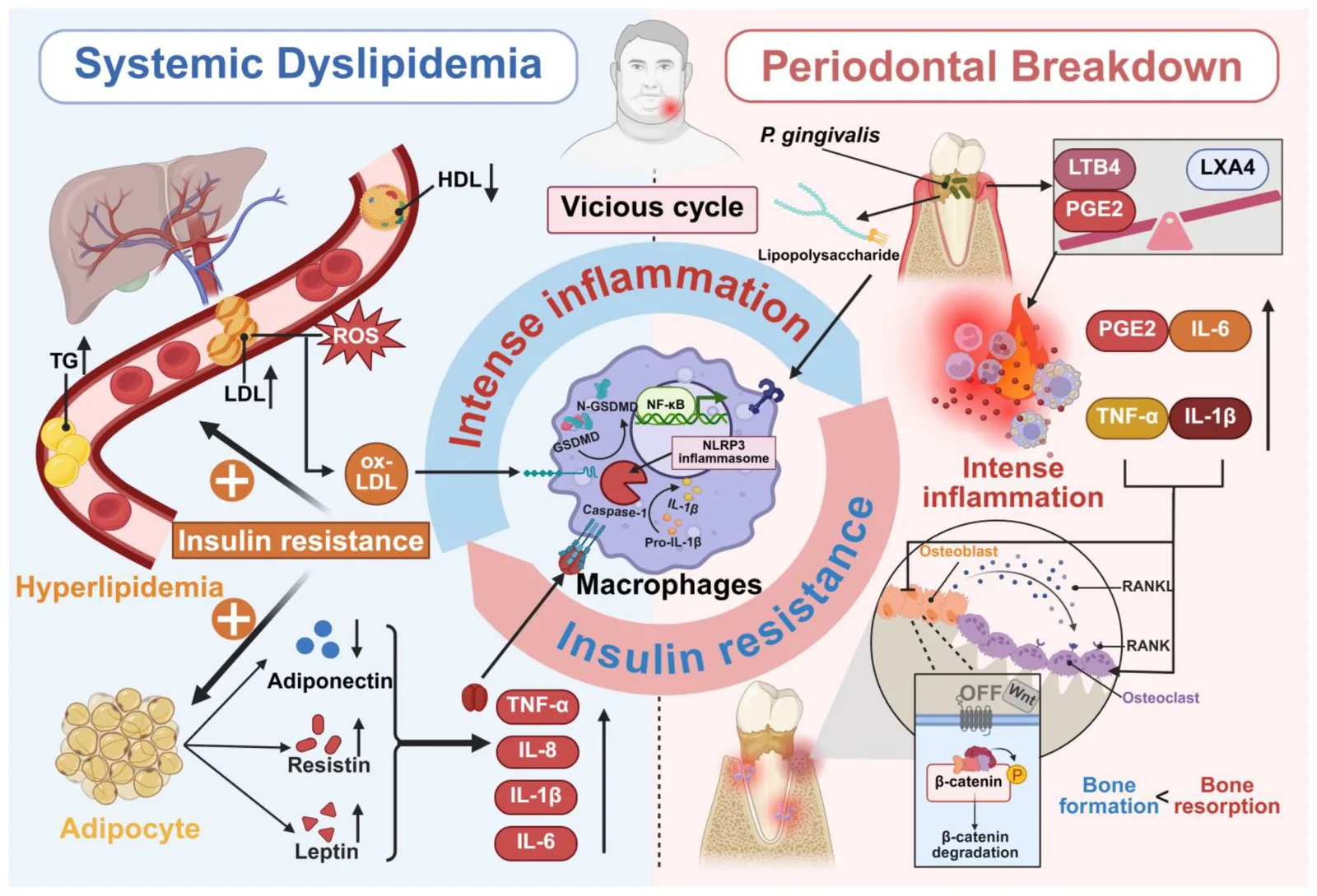
Proposed local lipid–redox–immune amplification in periodontitis. Altered lipid handling and oxidative stress may enhance inflammatory signaling and osteoclastogenic activity, while impaired specialized pro-resolving mediator pathways may delay inflammation resolution and favor continued alveolar bone loss. Most functional evidence supporting this framework remains preclinical. Created in BioRender. Yuxiao, Z. (2026) https://BioRender.com/sm2s84u (accessed on 31 August 2026).

Functional support comes primarily from ligature-induced periodontitis models. Soluble epoxide hydrolase inhibition increased SPM production and receptor expression, reduced osteolytic cytokine responses, and promoted a more resolving macrophage phenotype [72]. A subsequent study found that the same intervention preserved alveolar bone architecture and increased the ratio of regulatory T cells to Th17 cells, while soluble epoxide hydrolase expression in human gingiva correlated positively with disease severity [73]. Together, the evidence supports a restrained local model in which oxidative lipid injury, impaired resolution signaling, and dysregulated immune-cell activity prolong conditions favorable to osteolytic remodeling (Figure 3). The systemic direction and causality of the association are considered separately in Section 4.3.

### 4.3. Systemic Associations Between Periodontitis and Lipid Dysregulation

**Figure 4:**
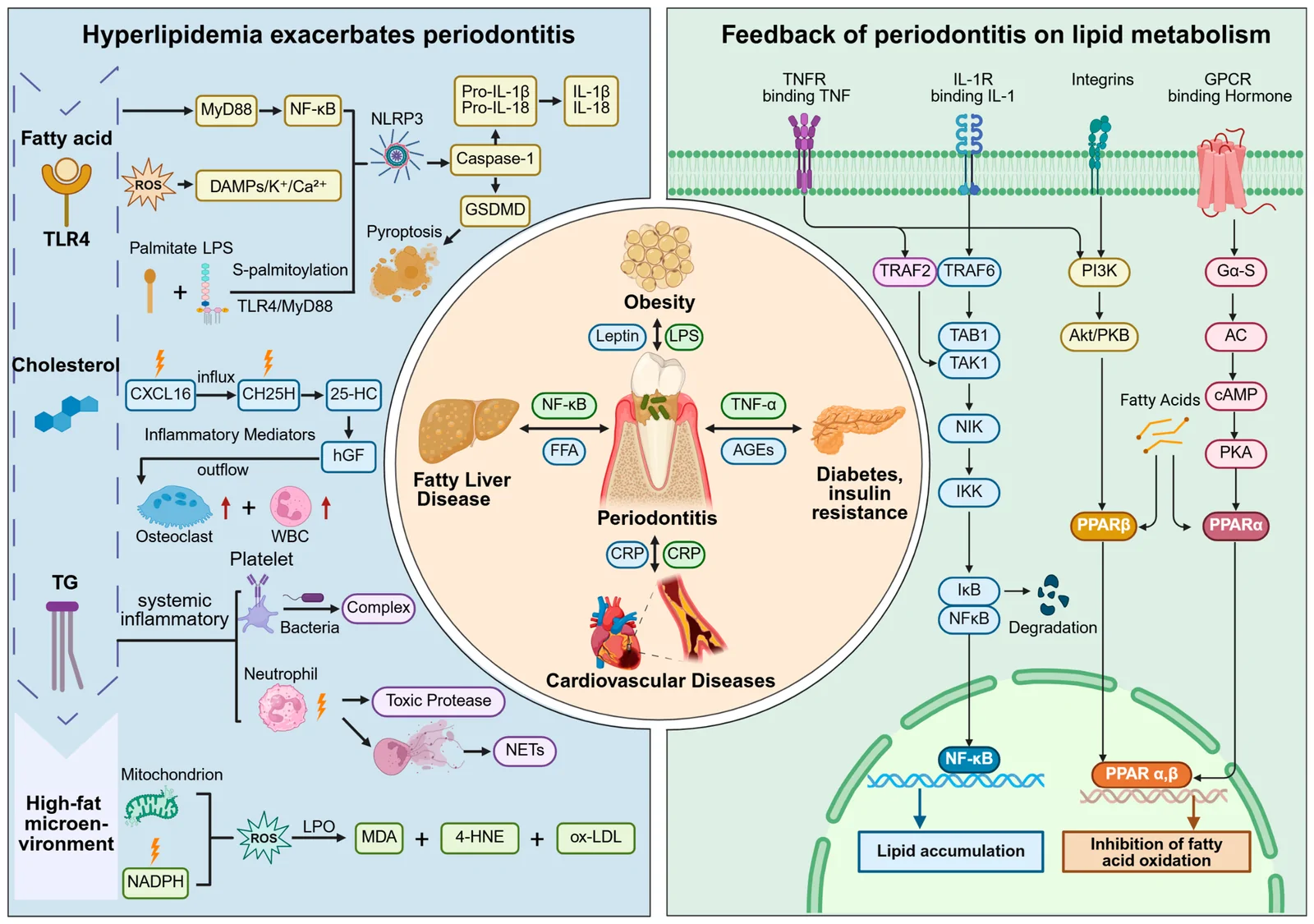
Proposed systemic associations between periodontitis and lipid dysregulation. Observational studies suggest that adverse metabolic profiles are associated with greater periodontal disease burden, whereas longitudinal findings link periodontal inflammation with subsequent changes in circulating lipids and lipoprotein composition. These relationships may be modified by obesity, insulin resistance, and glycemic control; intervention and Mendelian randomization evidence do not yet establish a fixed bidirectional causal cycle. Created in BioRender. Yuxiao, Z. (2026) https://BioRender.com/hi090yw (accessed on 31 August 2026).

At the systemic level, the relationship between periodontitis and dyslipidemia may be bidirectional (Figure 4): lipid excess may enhance periodontal inflammatory signaling, whereas periodontitis-associated inflammatory inputs may alter lipid handling and fatty-acid oxidation. However, the direction and magnitude of this association remain context-dependent. Population-based studies generally indicate that adverse metabolic profiles accompany greater periodontal disease burden. In the CHIEF Oral Health study of young adults, larger waist circumference and higher serum triglyceride concentrations were independently associated with localized stage II/III periodontitis, whereas total cholesterol and high-density lipoprotein cholesterol were not associated with disease in the adjusted analysis [74]. This heterogeneity across individual lipid measures is important: it suggests that periodontitis is unlikely to be related uniformly to all conventional lipid parameters. An updated systematic review and meta-analysis likewise found that periodontitis was associated overall with dyslipidemia, higher mean triglyceride, total cholesterol, and low-density lipoprotein cholesterol levels, and lower high-density lipoprotein cholesterol levels; however, substantial heterogeneity was present across the underlying observational studies [75]. Thus, the clinical literature supports an association between periodontal disease and systemic lipid disturbance, while also indicating that this association is influenced by participant characteristics, metabolic comorbidities, and study design.

Evidence for the reverse direction—namely, that periodontal inflammation may influence circulating lipoprotein metabolism—has emerged from longitudinal analyses. In the SHIP-TREND cohort, periodontal variables were associated with higher follow-up concentrations of triglycerides, total cholesterol, and low-density lipoprotein cholesterol over seven years, as well as with cholesterol-enriched apolipoprotein B-containing particles, including small dense low-density lipoprotein, very-low-density lipoprotein, and intermediate-density lipoprotein particles [76]. A systematic review of randomized controlled trials found no significant overall effects of periodontal therapy on total cholesterol, low-density lipoprotein cholesterol, high-density lipoprotein cholesterol, or triglycerides at six months [77].

Genetic evidence further argues against describing this relationship as a fixed causal loop. A bidirectional Mendelian randomization analysis did not identify significant causal effects between genetically predicted conventional serum lipid traits and periodontitis [78]. Rather than excluding a clinically relevant interaction, this discrepancy from observational and longitudinal findings may reflect heterogeneity in the underlying metabolic phenotype. Obesity and metabolic syndrome combine altered lipid handling with adiposity, insulin resistance, and low-grade inflammation; a systematic review and meta-analysis reported an overall association between obesity and periodontitis, although effect estimates varied substantially across populations and age groups [79]. Similarly, deep periodontal pockets were associated with an increased risk of incident metabolic syndrome in an 8-year cohort [80]. Diabetes is a further important modifier, as poor glycemic control is associated with greater periodontal risk and severity [81]. Collectively, current evidence supports a clinically relevant interplay between periodontal inflammation and systemic lipid homeostasis, the expression of which is shaped by the broader metabolic phenotype. Assessment of adiposity, metabolic syndrome components, and glycemic control may therefore help identify patients with greater susceptibility to progressive periodontal bone loss.

## 5. Oral Microbiota-Driven Lipid Metabolic Dysregulation and Impaired Bone Homeostasis

### 5.1. Periodontal Pathogen-Induced Lipid-Sensing Changes and Osteoclastogenic Bias

Periodontal pathogens can modify lipid handling in host myeloid cells, although the periodontal relevance of these observations depends on the cellular and experimental context. In RAW264.7 and THP-1 macrophages, infection with *Porphyromonas gingivalis* or *Fusobacterium nucleatum* increased neutral-lipid droplets and cholesteryl ester accumulation, together with alterations in the cholesterol-efflux regulators ABCG1 and CYP46A1 and enhanced Ca^2+^/ROS signaling [82]. A separate study further showed that *F. nucleatum* increased macrophage cholesterol uptake and reduced lipid efflux; its galactose-binding protein interacted with cyclophilin A and activated PI3K–AKT, MAPK, and NF-κB signaling, thereby promoting lipid deposition [83]. These findings support pathogen-associated reprogramming of macrophage lipid handling, but their principal endpoints were foam-cell formation and atherosclerosis-related responses rather than periodontal bone loss. At the periodontal tissue level, a high-fat-diet-induced metabolic-syndrome model combined with local LPS-induced experimental periodontitis showed that imipramine, a functional inhibitor of acid sphingomyelinase, attenuated the increase in osteoclast formation and alveolar bone loss [84]. In macrophages, imipramine also reduced the enhanced expression of IL-6, c-FOS, and several pro-inflammatory genes induced by combined palmitate and LPS exposure [84]. This result indicates that sphingolipid-related pathways can influence the extent to which a metabolically altered host responds to periodontal microbial challenge.

A more specific link between cholesterol sensing and periodontal bone resorption has been identified in osteoclast precursors. In a chronic *P. gingivalis* infection model, the frequency of CD11b^+^c-fms^+^Ly6C^hi^ osteoclast precursors increased in the bone marrow, spleen, and peripheral blood, and these cells displayed enhanced osteoclastogenic activity [85]. In a subsequent study, *P. gingivalis* infection reduced liver X receptor (LXR) expression and its downstream target *ApoE* in this precursor population [86]. In a ligature-induced periodontitis model, pharmacological LXR activation reduced local c-fms expression and attenuated periodontal bone loss [86]. Together, the infection and ligature models place cholesterol-responsive transcriptional control within the pathway linking inflammatory osteoclast precursor activity to periodontal bone resorption. Separately, extracellular vesicles from *P. gingivalis* and *Tannerella forsythia* promoted osteoclast differentiation mainly through TLR2 activation by bacterial lipoproteins and/or LPS [87]. This demonstrates a direct microbial route to osteoclast activation. Overall, periodontal pathogens may intensify bone resorption through both altered host lipid-sensing programs and direct microbial signaling; these mechanisms need to be tested concurrently in periodontal models.

### 5.2. Oral–Gut Axis: Translocation of Periodontal Bacteria, Gut Dysbiosis, and Systemic Metabolic–Inflammatory Activation

Periodontitis-associated oral–gut communication is supported by convergent human and experimental evidence. In a recent clinical multi-omics study, patients with periodontitis exhibited a gut microbial profile distinct from that of healthy participants, together with altered serum metabolite profiles; microbiota associated with periodontal health were relatively enriched in short-chain-fatty-acid-producing taxa [88]. Notably, periodontal therapy improved the salivary microbiota but did not substantially restore the gut microbial profile during follow-up, suggesting that intestinal ecological changes may not simply parallel local periodontal improvement. Experimental studies provide a temporal basis for this association. In obese diabetic mice, repeated oral administration of *P. gingivalis* resulted in the detection of *P. gingivalis*-specific peptides in fecal samples, altered gut microbial composition and intestinal metabolites, and aggravated hyperglycaemia [89]. Thus, repeated exposure to periodontal pathogens may influence the intestinal ecosystem through the digestive tract, while changes in microbial composition and metabolite availability provide a plausible interface between periodontal dysbiosis and systemic metabolic disturbance.

The metabolic relevance of this axis is supported by studies that experimentally manipulated the gut microbiota. In mice with experimental periodontitis, reduced abundances of several butyrate-producing taxa were accompanied by elevated fasting glucose, HbA1c, and glucose intolerance; gut-microbiota depletion or cohousing with healthy mice improved these metabolic abnormalities and reduced circulating endotoxin and IL-6 levels [90]. In *ApoE*−/− mice, ligature-induced periodontitis altered gut microbial composition, increased colonic and systemic inflammatory markers, and shifted tibial bone remodeling toward increased osteoclast activity and a higher RANKL/OPG ratio. Importantly, transplantation of microbiota from periodontitis donors partially reproduced colonic and skeletal phenotypes in antibiotic-treated recipients [91]. Evidence also indicates that intestinal dysbiosis can feed back on periodontal tissues: antibiotic-induced microbiota disruption increased alveolar bone loss and periodontal Th17-related responses in experimental periodontitis, whereas fecal microbiota transplantation alleviated these changes [92]. These findings position the oral–gut axis as a metabolic and immunological amplifier that can connect periodontal dysbiosis with systemic metabolic inflammation and altered bone homeostasis.

### 5.3. Dual Roles of Oral Microbe-Derived Short-Chain Fatty Acids: Local Tissue Damage Versus Systemic Protection

Among the microbial metabolites that may connect oral–gut ecological changes with periodontal bone remodeling, short-chain fatty acids (SCFAs) deserve particular attention. Their biological effects cannot be inferred from molecular identity alone, because the source, concentration, site of exposure, and target-cell context differ markedly between periodontal pockets and the systemic circulation. Within periodontal pockets, SCFAs represent locally enriched microbial metabolites. Measurements of gingival crevicular fluid and saliva have shown that SCFA profiles differ according to periodontal disease severity, supporting their use as markers of the local dysbiotic microenvironment [93]. This local metabolite milieu can directly affect the epithelial barrier. In a three-dimensional gingival epithelial–connective tissue model, butyrate exposure induced epithelial cell death and the release of damage-associated molecular patterns, including extracellular DNA and SAP130 [94]. Consistent with this concept, a recent microbiome-metabolite study identified propionate as a product of periodontal disease-associated bacteria; propionate inhibited the growth of human gingival epithelial cells and increased IL-8 expression [95]. Thus, locally accumulated SCFAs may weaken epithelial containment and increase the inflammatory burden within periodontal tissues, creating conditions that favor destructive rather than regenerative remodeling.

**Figure 5:**
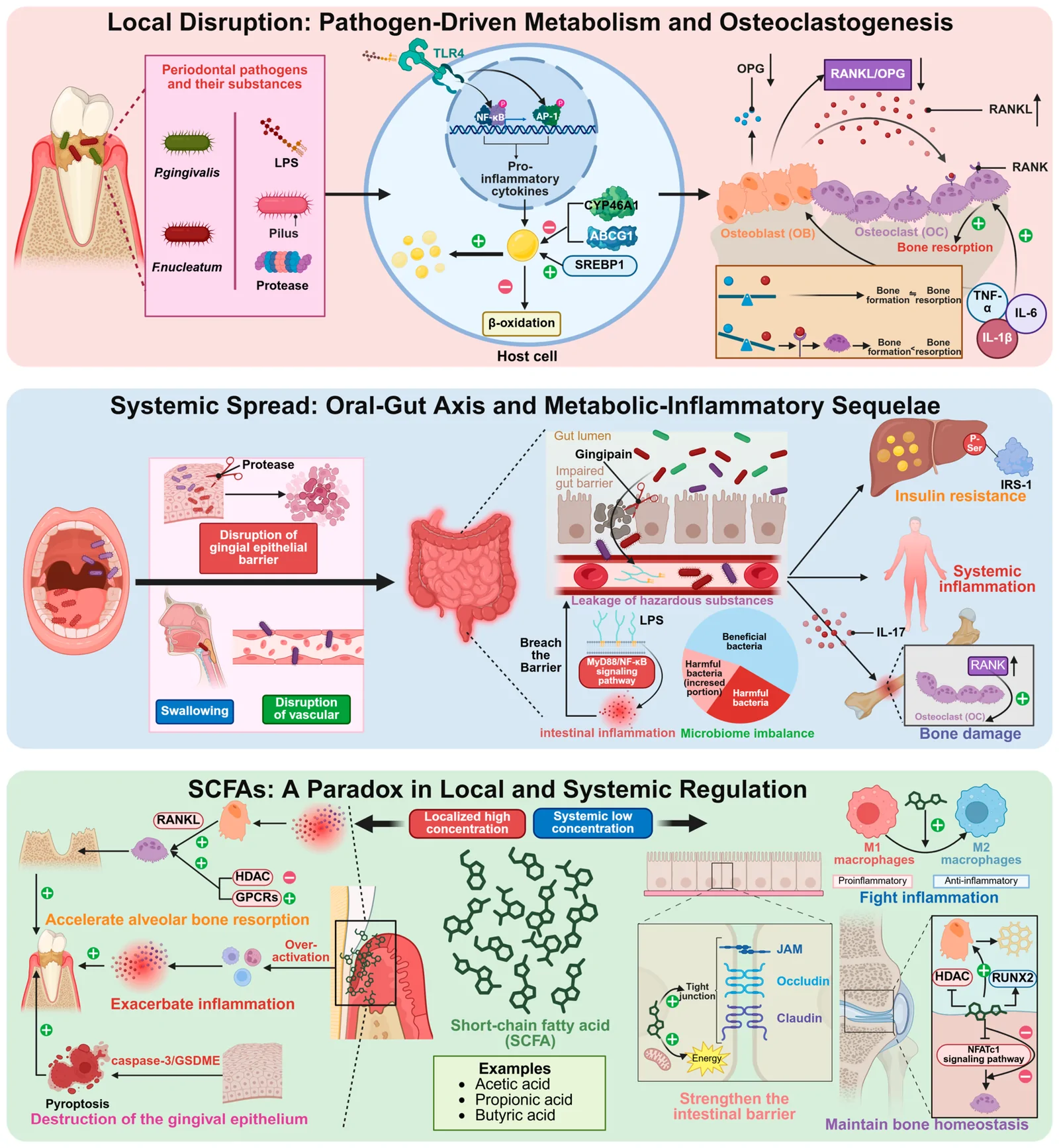
Oral microbiota influence bone homeostasis at both local and systemic levels through metabolic reprogramming and immunoinflammatory regulation. Periodontal pathogens perturb local lipid metabolism and promote bone resorption via RANKL/OPG imbalance. Through the oral–gut axis, they induce systemic meta-inflammation, indirectly disrupting bone metabolism. Microbial metabolite SCFAs exert dual effects: high local levels exacerbate tissue damage, while systemic levels are bone-protective. Created in BioRender. Yuxiao, Z. (2026) https://BioRender.com/skqhu1p (accessed on 31 August 2026).

In contrast, SCFAs generated in the intestinal ecosystem or administered through systemic metabolic interventions can influence bone cells in a different exposure context. In a mouse model of inflammatory arthritis, SCFA supplementation modified the transcriptional profile of osteoclast precursors, inhibited osteoclast differentiation at serum-relevant concentrations, and alleviated osteoclast-mediated osteoporosis [96]. Acetate and propionate also directly enhanced early osteoblastic differentiation in osteoblastic cells, whereas butyrate did not produce the same effect under the tested conditions [97]. Importantly, a recent study of diabetic periodontitis reported that oral butyrate administration reduced alveolar bone loss while suppressing macrophage M1 polarization and PANoptosis-like cell death in periodontal tissues [98]. These findings indicate that SCFA effects are determined by molecular species, concentration, exposure route, target cell population, and inflammatory background. High local concentrations of periodontal microbial metabolites may compromise barrier function, whereas microbiota-associated systemic SCFA signals may support immunometabolic balance and restrain pathological bone resorption (Figure 5).

## 6. Therapeutic Interventions for Disorders of Lipid Metabolism and Periodontal Bone Preservation

Conventional periodontal therapy remains based on plaque control and mechanical debridement. Lipid-targeted interventions should therefore be considered adjunctive rather than substitutive treatments. Their clinical maturity differs markedly. Human evidence is available mainly for locally delivered statins and several nutritional or natural adjuncts, whereas PPARγ modulation, responsive biomaterials, and metabolic conditioning combined with stem cell therapy remain largely preclinical. Moreover, most clinical studies report inflammatory or soft-tissue periodontal outcomes rather than direct alveolar bone preservation. This section therefore evaluates therapeutic strategies according to evidence level and distinguishes demonstrated periodontal effects from indirect or conceptual links to lipid metabolism.

### 6.1. Clinically Evaluated Adjunctive Interventions

#### 6.1.1. Statin Therapy: Local and Systemic Evidence

Statins may influence periodontal tissues through two distinct routes. Systemic statins are primarily prescribed to treat dyslipidemia and reduce cardiovascular risk, whereas locally delivered statins have been investigated as adjuncts to conventional periodontal treatment. The strength and clinical meaning of the evidence differ between these two routes.

Local statin delivery currently has the stronger and more direct periodontal evidence. In a double-masked randomized trial of 50 patients with Stage III, Grade B periodontitis, locally applied simvastatin produced greater 12-month improvements in clinical attachment level, probing depth, and bleeding on probing than the control treatment [99]. However, the trial did not demonstrate significant interproximal bone gain. A separate short-term study of an atorvastatin-loaded cubosome gel reported reductions in probing depth, bleeding index, plaque index, and gingival TGF-β1 levels [100]. Because the formulation also contained eugenol and nanocarriers, its effects cannot be attributed to atorvastatin alone.

Systemic statins may also provide periodontal benefits through their lipid-lowering and anti-inflammatory effects. In a randomized pilot study of 38 patients with chronic periodontitis, oral atorvastatin at 20 mg/day for three months, combined with mechanical periodontal treatment, improved serum lipid levels, tooth mobility, and a radiographic measure of alveolar bone loss compared with placebo [101]. However, the study was small and short-term, and the wider systemic evidence remains limited and inconsistent.

Overall, local statin delivery provides more direct evidence of adjunctive periodontal benefit, whereas the independent periodontal effect of systemic statin therapy remains uncertain. Future trials should directly compare local and systemic administration and include standardized three-dimensional alveolar bone measurements, longer follow-up, and systematic safety assessment.

#### 6.1.2. Exploratory PPARγ-Directed Therapy

Human evidence is limited to a small exploratory study in patients with Stage II, Grade B periodontitis. Adding pioglitazone to standard periodontal treatment according to a proposed circadian schedule improved several periodontal indices [102]. However, each treatment group included only 12 patients, and alveolar bone changes were not directly assessed.

This isolated study is insufficient to establish the periodontal efficacy of systemic PPARγ activation. It also does not demonstrate that pioglitazone can simultaneously correct dyslipidemia and preserve periodontal bone. PPARγ agonists should therefore not be recommended specifically for periodontitis without larger controlled trials, direct bone measurements, and systematic safety assessment.

#### 6.1.3. Nutritional and Natural Adjuncts

Human studies have evaluated resveratrol, Mediterranean dietary patterns, omega-3 polyunsaturated fatty acids, and probiotics as adjuncts to conventional periodontal treatment. These interventions may influence periodontal inflammation, but their effects on lipid metabolism and alveolar bone should be evaluated separately.

Clinical evidence for resveratrol is mixed. A randomized study of 160 patients with aggressive periodontitis reported improved periodontal indices and lower local and systemic inflammatory markers after oral supplementation [103]. By contrast, a double-blind trial of 40 patients found no significant improvements in probing depth, clinical attachment loss, bleeding, or salivary IL-1β and IL-8 after four weeks; only the plaque index improved [104]. Differences in patient populations, dosage, background treatment, and follow-up may explain these conflicting results.

A randomized trial involving 42 patients with gingivitis found that a six-week Mediterranean diet reduced gingival inflammation, particularly bleeding on probing, without changing plaque levels [105]. However, the participants did not have periodontitis, and alveolar bone was not assessed. Clinical evidence is more directly relevant for omega-3 fatty acids. In a randomized trial of 30 patients with periodontitis, omega-3 supplementation combined with nonsurgical periodontal therapy produced greater improvements in probing depth and clinical attachment level than periodontal therapy with a soybean-oil placebo, although bleeding outcomes did not differ [106]. A meta-analysis of ten randomized trials also found modest additional improvements in probing depth and clinical attachment level [107]. However, the included studies used different doses and formulations and did not directly demonstrate alveolar bone regeneration.

The effects of probiotics appear to be strain- and protocol-dependent. In a randomized trial of 30 patients, yogurt containing *Bifidobacterium animalis* improved plaque, gingival inflammation, and bleeding after nonsurgical periodontal treatment but did not provide additional improvements in probing depth or clinical attachment level [108]. Similarly, a double-blind trial of 28 patients receiving supportive periodontal therapy found that daily *Lactobacillus reuteri* reduced bleeding on probing, whereas the other clinical outcomes showed no consistent benefit [109]. Neither study assessed serum lipid profiles or alveolar bone changes.

Taken together, these nutritional and natural interventions may improve selected inflammatory or soft-tissue periodontal outcomes, but the effects are generally modest and inconsistent. Current human evidence does not establish predictable alveolar bone preservation, bone regeneration, or correction of dyslipidemia. These approaches should therefore be regarded as possible adjuncts rather than established lipid-targeted periodontal treatments.

### 6.2. Preclinical Metabolic and Pharmacological Strategies

Preclinical studies provide mechanistic support for metabolic intervention, but findings from cell cultures and animal models should not be interpreted as evidence of clinical efficacy.

#### 6.2.1. PPARγ Modulation

PPARγ links lipid metabolism with macrophage activity and periodontal inflammation. However, its effects depend on the target cell, dose, metabolic state, and treatment duration, making broad activation or inhibition difficult to predict.

In rats with experimental periodontitis, rosiglitazone reduced the M1/M2 macrophage ratio, IL-1β expression, osteoclast numbers, and alveolar bone loss [110]. A separate study showed that FoxO1 inhibition enhanced PPARγ-related signaling, promoted anti-inflammatory macrophage polarization, and partly restored osteogenic activity under inflammatory conditions. In the same study, rosiglitazone also reduced inflammation in a ligature-induced periodontitis model [111].

Together, these findings suggest that the periodontal benefit of PPARγ activation may arise mainly from macrophage reprogramming and reduced inflammation. However, these studies do not demonstrate that systemic PPARγ activation can simultaneously correct dyslipidemia and preserve periodontal bone. Because PPARγ also regulates the balance between adipogenic and osteogenic differentiation, broad or prolonged activation may produce different effects in macrophages and mesenchymal progenitor cells. Future studies should therefore prioritize cell-specific, temporary, or locally controlled modulation and should measure lipid changes and three-dimensional alveolar bone outcomes directly.

#### 6.2.2. Curcumin and Other Experimental Interventions

Evidence for curcumin remains preclinical. In rats with experimental periodontitis, local curcumin combined with scaling and root planing reduced inflammatory-cell infiltration, osteoclast numbers, and alveolar bone resorption [112]. However, these findings mainly support an anti-inflammatory and anti-resorptive effect and do not demonstrate that curcumin directly corrects lipid metabolism.

Preclinical evidence also suggests that dietary lipid composition may influence periodontal bone responses. In a mouse model, an oleic acid-enriched diet increased circulating pro-resolving lipid mediators and improved alveolar trabecular bone after *Porphyromonas gingivalis* inoculation [113]. However, crestal bone loss remained unchanged. The improvement in trabecular bone should therefore not be interpreted as complete protection against periodontitis-associated alveolar bone loss.

Together, these studies suggest that curcumin and oleic acid may reduce inflammation or modify selected bone outcomes in experimental models. Nevertheless, their mechanisms and endpoints differ, and neither intervention has demonstrated predictable periodontal bone preservation or correction of dyslipidemia in patients. Further studies should include direct lipid measurements, standardized periodontal bone outcomes, and comparison with conventional periodontal treatment.

### 6.3. Emerging Biomaterial-Based Delivery

Biomaterial-based delivery systems may improve local drug retention, reduce systemic exposure, and combine anti-inflammatory, antimicrobial, and osteogenic effects. Among the available systems, those tested in metabolically compromised periodontal models or delivering agents with a direct lipid-related rationale are most relevant to this review. Nevertheless, current evidence remains entirely preclinical.

In a high-fat-diet-induced rat model, a chitosan-based hydrogel containing granulocyte–macrophage colony-stimulating factor and resveratrol, used together with scaling and root planing, reduced periodontal inflammation and tissue destruction and shifted the local T-cell response toward an anti-inflammatory state [114]. Because this system was evaluated under combined metabolic and periodontal stress, it provides more disease-specific evidence than biomaterials tested only in conventional periodontal defects. However, the study did not establish normalization of systemic or local lipid metabolism.

Local simvastatin delivery has also shown benefit in animal models. A bone-targeted thermoresponsive hydrogel preserved alveolar bone and reduced periodontal inflammation in ligature-induced periodontitis [115]. Similarly, a thermoresponsive polymeric simvastatin prodrug increased periodontal bone volume and reduced neutrophil infiltration and IL-1β secretion compared with free simvastatin acid [116]. These findings support the value of controlled local delivery but do not demonstrate clinical efficacy.

Other systems combine simvastatin with regenerative or antimicrobial agents. A 3D-printed bilayer membrane containing grape seed extract and simvastatin provided sustained drug release, antibacterial activity, and osteogenic support and enhanced alveolar bone formation in an animal periodontal-defect model [117]. PLGA-chitosan nanospheres carrying simvastatin and doxycycline also reduced IL-1β and MMP-8 expression and limited bone loss in rats with experimental periodontitis [118]. Because these formulations contain multiple active components, their effects cannot be attributed to lipid modulation or simvastatin alone.

None of these biomaterial systems has been validated in patients. Differences in animal models, defect types, drug doses, delivery systems, and outcome measures also limit direct comparison. Before clinical translation, future studies should assess long-term retention, degradation, local toxicity, systemic exposure, and manufacturing consistency. Studies claiming metabolic regulation should measure local and systemic lipid changes together with standardized three-dimensional periodontal bone outcomes.

### 6.4. Metabolic Conditioning of Stem Cells: Preclinical Rationale

Clinical studies suggest that stem cell therapy itself may provide modest benefits for periodontal regeneration. A meta-analysis of five randomized trials involving 118 patients reported improvements in clinical attachment level, probing depth, and intrabony defect depth compared with cell-free treatment [119]. However, the included studies were small and methodologically heterogeneous. More importantly, they evaluated stem cell therapy alone rather than stem cell therapy combined with lipid-modulating interventions. These findings therefore provide clinical context for periodontal regeneration but not clinical evidence for metabolic conditioning.

Preclinical studies indicate that the surrounding lipid environment can strongly influence periodontal stem cell function. In cultured human periodontal ligament stem cells, palmitic acid impaired osteogenic differentiation by suppressing the *AC018926.2*–ITGA2/FAK/AKT pathway [45]. By contrast, periodontal ligament stem cells can produce the pro-resolving lipid mediator maresin conjugate in tissue regeneration 3 (MCTR3). In pig periodontal ligament stem cells, docosahexaenoic acid increased MCTR3 synthesis, while MCTR3 reduced inflammatory cytokine production under inflammatory conditions [120]. These findings suggest that lipid-related signals may either impair or support periodontal stem cell activity, depending on the lipid mediator and cellular environment. However, both studies were conducted in cell cultures and did not test periodontal regeneration after cell transplantation.

PPARγ modulation further illustrates this context dependence. In insulin-resistant periodontal ligament stem cells, rosiglitazone increased insulin sensitivity and migration while reducing oxidative stress and inflammation and preserving osteogenic and odontogenic markers [121]. By contrast, excessive PPARγ signaling in bone marrow mesenchymal stem cells favored adipogenic differentiation and impaired osteogenesis, whereas suppression of PPARγ partly restored the osteogenic–adipogenic balance [122]. These findings are not necessarily contradictory because they involve different cell populations and metabolic conditions. They indicate that broad PPARγ activation or inhibition may be less suitable than cell-specific, temporary, or locally controlled modulation.

At present, no clinical study has tested stem cell transplantation combined with statins, PPARγ modulators, or lipid-targeted nanoparticles in periodontitis. Such combinations remain experimental. Future studies should identify the appropriate cell source and treatment window, confirm local and systemic safety, and measure stem cell survival, lipid changes, and three-dimensional periodontal bone regeneration.

### 6.5. Translational Limitations and Research Priorities

Several gaps still limit clinical translation. Most human studies were small and focused on probing depth, clinical attachment, bleeding, or inflammatory markers rather than direct changes in alveolar bone. Local statins currently have the clearest clinical support, while evidence for systemic statins, PPARγ agonists, dietary interventions, and natural products is less consistent. Biomaterials and metabolic conditioning of stem cells remain largely preclinical. Comparisons across studies are also difficult because of differences in patient populations, animal models, doses, delivery routes, and follow-up periods. In addition, many studies proposed metabolic effects without directly measuring lipid changes.

**Figure 6:**
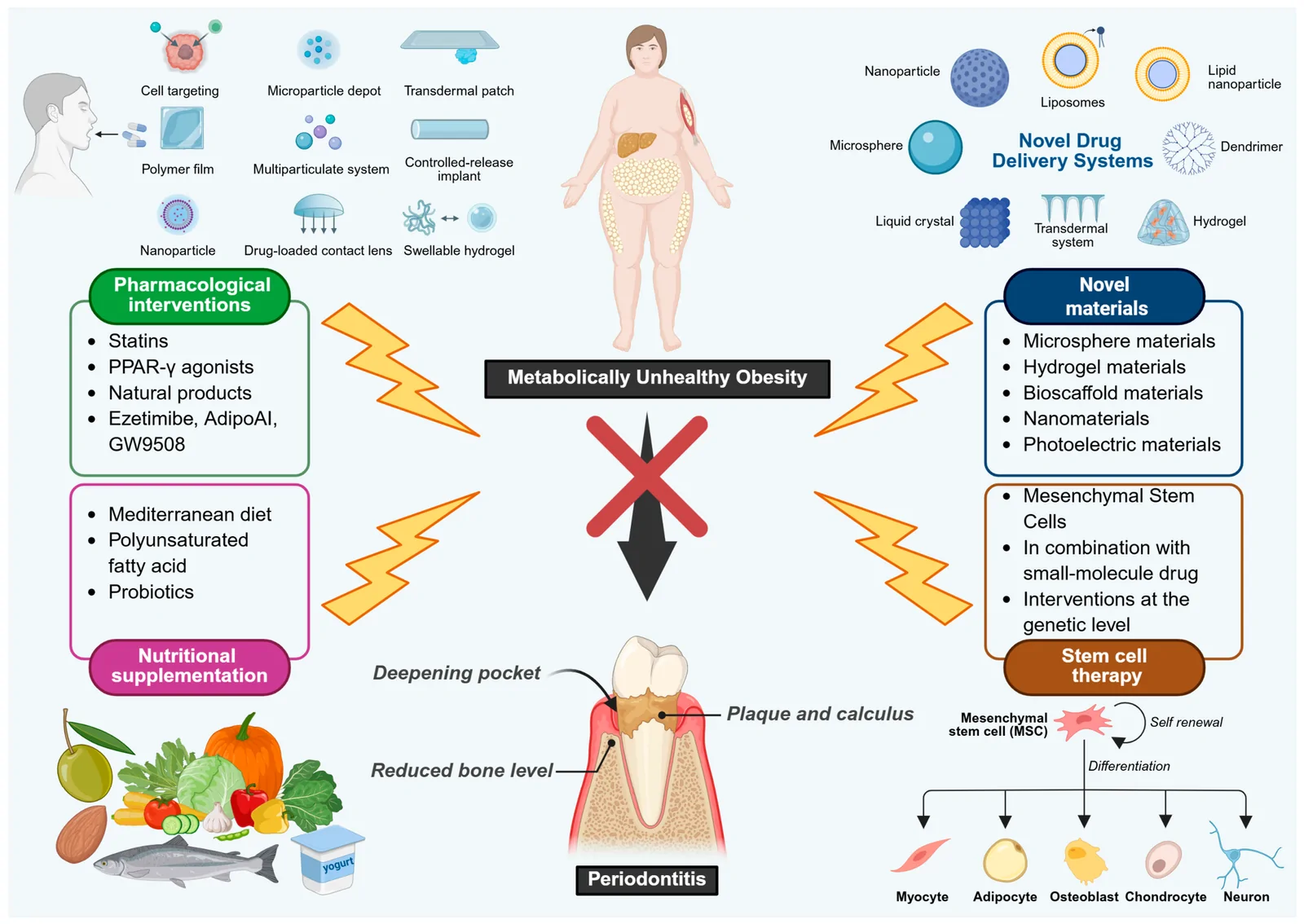
Therapeutic interventions targeting lipid dysregulation in periodontitis. Schematic illustration of four strategies to disrupt the dyslipidemia–periodontitis cycle: (1) pharmacological therapy, including statins and PPARγ agonists; (2) nutritional regulation, such as the Mediterranean diet and probiotics; (3) biomaterial-based delivery systems, including drug-loaded hydrogels; (4) stem cell therapy. Collectively, these integrated approaches offer targeted interventions for the management of metabolic–periodontal bone destruction. Created in BioRender. Yuxiao, Z. (2026) https://BioRender.com/gtn18le (accessed on 31 August 2026).

Future trials should record patients’ lipid status, obesity, diabetes, and medication use and should measure lipid profiles, periodontal inflammation, clinical indices, and three-dimensional bone changes together. Larger samples, longer follow-up, and careful safety monitoring are also needed. Until stronger evidence is available, lipid-related treatments should be viewed as adjuncts to conventional periodontal care rather than replacements for it. The principal therapeutic strategies targeting lipid dysregulation in periodontitis are summarized in Figure 6.

## 7. Conclusions

Current evidence suggests that lipid metabolic reprogramming modifies the balance between bone formation and resorption in periodontal tissues. Normal lipid utilization supports osteoblast differentiation, osteogenic signaling, and stem-cell-mediated regeneration, ensuring a balanced relationship between bone formation and resorption. When lipid homeostasis is disrupted, however, elevated free fatty acids, oxidized lipids, and cholesterol excess may increase oxidative stress, activate NF-κB and MAPK signaling, and promote pro-inflammatory immune responses. These changes may enhance osteoclastogenesis, inhibit osteoblast function, impair the osteogenic potential of PDLSCs and BMSCs, and thereby contribute to alveolar bone loss. Multiple signaling pathways—including PPARγ, Wnt/β-catenin, AMPK-mTOR, Notch, leptin, and BMP—mediate the crosstalk between lipid disturbances and periodontal destruction. Their effects are not uniform, however, and may vary with cell type, metabolic conditions, and exposure duration, as illustrated by the dual effects of PPARγ. However, most mechanistic evidence comes from cell and animal studies, while human studies mainly demonstrate associations. High-fat-diet models are also influenced by obesity, insulin resistance, and microbiome changes. Lipid dysregulation should therefore be regarded as a potential modifier rather than a confirmed independent cause of periodontal bone loss in humans. Among the available interventions, locally delivered statins currently have the clearest clinical support, whereas most other lipid-targeted strategies remain preclinical. Future studies should determine whether these approaches can safely complement conventional periodontal treatment and improve bone regeneration.

## Figures and Tables

**Table 1:** Lipid-related signaling pathways in periodontal bone remodeling.

Pathway	Target	Lipid Metabolism Effect	Bone Remodeling Effect	Role in Periodontitis	Therapeutic Potential	Reference
PPARγ	H3K27ac, Wnt/β-catenin, GSK3β, BMP2, OPG/RANKL	Links lipid sensing to the osteogenic–adipogenic switch; its effects vary with cell state and exposure duration.	Maintains osteogenic and cementogenic gene expression in periodontal ligament fibroblasts, but sustained activation suppresses *RUNX2* and *BMP2* and shifts OPG/RANKL signaling toward bone resorption.	Early activation reduces inflammation and bone loss, whereas prolonged activation may impair periodontal regeneration	Short-term or cell-specific modulation may control inflammation while avoiding the adverse skeletal effects of prolonged activation	[54,55,56]
Wnt/β-catenin	PPARγ, GSK3β, β-catenin, RUNX2, BMP2, lysine lactylation	Counterbalances PPARγ-associated adipogenic commitment and integrates metabolic and lactylation signals.	Promotes PDLSC osteogenesis and cellular cementum apposition.	Inflammation suppresses Wnt activity, whereas its restoration rescues PDLSC osteogenesis.	Restoring Wnt activity under inflammatory conditions may enhance PDLSC osteogenesis and cementum regeneration.	[57,58]
AMPK-mTOR	ROS, AMPK, mTOR, MCT1, autophagy, HMGB1	Nutrient stress activates AMPK and suppresses mTOR to induce adaptive autophagy, whereas excess lactate activates MCT1–mTOR signaling and reduces autophagy.	Early autophagy supports cellular adaptation. Lactate-induced mTOR activation inhibits PDLSC osteogenesis, while rapamycin partly restores it; metformin reduces oxidative stress and alveolar bone resorption.	Dysregulated energy sensing links metabolic and oxidative stress to impaired regeneration and periodontal bone loss.	AMPK activation or controlled mTOR inhibition may restore autophagy, reduce oxidative stress, and support periodontal regeneration	[59,60,61]
Notch	Jagged1, NOTCH2, MSX2, COL1A1, OPG/RANKL, BMP	Direct regulation of periodontal lipid metabolism by Notch has not been demonstrated.	Jagged1–NOTCH2 promotes periodontal ligament cell mineralization but also lowers OPG, potentially favoring osteoclast formation. Canonical Notch is also required for BMP-induced osteogenesis in human mesenchymal stem cells.	Its effects are context-dependent: Notch may promote stromal mineralization while simultaneously enhancing osteoclastogenesis through reduced OPG.	Stage- and cell-specific modulation may enhance mineralization and BMP responses, whereas broad activation could increase osteoclastogenesis.	[62,63,64]
Leptin	Leptin receptor, OPG/RANKL, CCRL2, SFRP1, Wnt/β-catenin	Provides a systemic adipokine signal, but periodontal effects depend on leptin concentration; leptin-receptor expression alone does not demonstrate active leptin signaling.	Low leptin increases the OPG/RANKL ratio, whereas high leptin produces the opposite effect. In leptin-receptor-positive alveolar stromal cells, CCRL2–SFRP1 signaling suppresses Wnt and impairs bone healing.	May protect or impair periodontal bone depending on leptin concentration and inflammatory status; direct causal evidence remains limited.	Normalizing leptin exposure or blocking CCRL2–SFRP1-mediated Wnt inhibition may improve alveolar bone healing.	[66,67]
BMP	BMP2, BMP6, BMP9, Notch, NELL1, p38/ERK	Direct effects of pathological lipid exposure on periodontal BMP signaling remain unclear; BMP intersects with PPARγ–Wnt regulation of cell fate.	Notch supports BMP2/6-induced osteogenesis, while BMP9 promotes osteogenesis in inflamed PDLSCs. NELL1 further enhances BMP9-induced bone formation through p38/ERK signaling.	Its regenerative effect depends on Notch activity and the inflammatory environment; BMP9 alone does not clearly suppress inflammation.	Combining BMP9 with NELL1 or coordinated Notch modulation may enhance regeneration while limiting inflammatory effects.	[64,65]

## Data Availability

No new data were created or analyzed in this study. Data sharing is not applicable to this article.

## Abbreviations

The following abbreviations are used in this manuscript:

4-HNE	4-hydroxy-2-nonenal
25-HC	25-hydroxycholesterol
ABCG1	ATP-binding cassette subfamily G member 1
ABMMSCs	alveolar bone marrow mesenchymal stem cells
AC	adenylyl cyclase
ACC	acetyl-CoA carboxylase
AGEs	advanced glycation end products
AKT/PKB	protein kinase B
ALK	activin receptor-like kinase
ALP	alkaline phosphatase
AMPK	AMP-activated protein kinase
AP-1	activator protein 1
ApoE	apolipoprotein E
ATGL	adipose triglyceride lipase
ATP	adenosine triphosphate
BMP (BMP2/6/9)	bone morphogenetic protein (isoforms 2, 6, and 9)
BMSCs	bone marrow mesenchymal stem cells
BSP	bone sialoprotein
cAMP	cyclic adenosine monophosphate
CCRL2	C-C motif chemokine receptor-like 2
CD11b	cluster of differentiation 11b
c-fms	colony-stimulating factor 1 receptor (CSF1R)
c-FOS	Fos proto-oncogene, AP-1 transcription factor subunit
CH25H	cholesterol 25-hydroxylase
CHIEF	Cardiorespiratory Fitness and Hospitalization Events in Armed Forces study
COL1A1	collagen type I alpha 1 chain
CPT2/Cpt2	carnitine palmitoyltransferase 2 (human/mouse nomenclature, respectively)
CRP	C-reactive protein
CSF-1	colony-stimulating factor 1
CTSK	cathepsin K
CXCL16	C-X-C motif chemokine ligand 16
CYP46A1	cytochrome P450 family 46 subfamily A member 1 (cholesterol 24-hydroxylase)
DAMPs	damage-associated molecular patterns
DFCs	dental follicle cells
DFPCs	dental follicle precursor cells
DHA	docosahexaenoic acid
DNA	deoxyribonucleic acid
DPSCs	dental pulp stem cells
EPA	eicosapentaenoic acid
ERK	extracellular signal-regulated kinase
EZH2	enhancer of zeste 2 polycomb repressive complex 2 subunit
FAK	focal adhesion kinase
FFA	free fatty acid
FFAR2	free fatty acid receptor 2
FOXO1	forkhead box O1
FTO	fat mass and obesity-associated protein
Gαs	stimulatory G-protein alpha subunit
GMSCs	gingiva-derived mesenchymal stem cells
GPCRs	G-protein-coupled receptors
Grb2	growth factor receptor-bound protein 2
GSDMD	gasdermin D
GSDME	gasdermin E
GSH	reduced glutathione
GSK3β	glycogen synthase kinase 3 beta
H3K27ac	histone H3 lysine 27 acetylation
HbA1c	glycated hemoglobin A1c
HDAC	histone deacetylase
HDL	high-density lipoprotein
hGF	human gingival fibroblast
HMGB1	high-mobility group box 1
HMGCR	3-hydroxy-3-methylglutaryl-coenzyme A reductase
IκB	inhibitor of nuclear factor kappa B
IKK	IκB kinase
IL-1β	interleukin-1 beta
IL-1R	interleukin-1 receptor
IL-6	interleukin-6
IL-8	interleukin-8
IL-17	interleukin-17
IRS-1	insulin receptor substrate 1
ITGA2	integrin subunit alpha 2
JAK	Janus kinase
JAM	junctional adhesion molecule
JNK	c-Jun N-terminal kinase
LDL	low-density lipoprotein
LDLR	low-density lipoprotein receptor
LepR	leptin receptor
lncRNA	long non-coding RNA
LOX-1	lectin-like oxidized low-density lipoprotein receptor 1
LPS	lipopolysaccharide
LRP5	low-density lipoprotein receptor-related protein 5
LTB_4_	leukotriene B4
LXA_4_	lipoxin A4
LXR	liver X receptor
Ly6C^hi	lymphocyte antigen 6 complex, locus C1, high expression
M1/M2	pro-inflammatory/anti-inflammatory macrophage phenotypes
MAPK	mitogen-activated protein kinase
MCT1	monocarboxylate transporter 1
MCTR3	maresin conjugate in tissue regeneration 3
MDA	malondialdehyde
MG-GMSCs	marginal gingiva-derived mesenchymal stem cells
MMP-8	matrix metalloproteinase 8
mRNA	messenger RNA
MSCs	mesenchymal stem cells
MSX2	msh homeobox 2
mTOR	mechanistic target of rapamycin
MyD88	myeloid differentiation primary response 88
NAD+	oxidized nicotinamide adenine dinucleotide
NADPH	reduced nicotinamide adenine dinucleotide phosphate
NELL1	neural EGFL-like 1
NETs	neutrophil extracellular traps
NF-κB	nuclear factor kappa B
NFATc1	nuclear factor of activated T cells, cytoplasmic 1
N-GSDMD	N-terminal fragment of gasdermin D
NIK	NF-κB-inducing kinase
NLRP3	NLR family pyrin domain containing 3
NOTCH2	notch receptor 2
NRF2	nuclear factor erythroid 2-related factor 2
OB	osteoblast
OC	osteoclast
OCN	osteocalcin
OPG	osteoprotegerin
ox-LDL	oxidized low-density lipoprotein
PDLSCs	periodontal ligament stem cells
PGE_2_	prostaglandin E2
PI3K	phosphatidylinositol 3-kinase
PKA	protein kinase A
PLGA	poly (lactic-co-glycolic acid)
PPARα/β/γ (PPARγ2)	peroxisome proliferator-activated receptor alpha/beta/gamma (gamma isoform 2)
P-Ser	phosphoserine
PUFAs	polyunsaturated fatty acids
RANK	receptor activator of nuclear factor-κB
RANKL	receptor activator of nuclear factor-κB ligand
RAW264.7	murine macrophage cell line RAW 264.7
ROS	reactive oxygen species
RUNX2	RUNX family transcription factor 2
RvE1	resolvin E1
S1P	sphingosine-1-phosphate
S1P/S2P (proteases)	site-1/site-2 proteases; Figure 1 only
SAP130	Sin3A-associated protein 130
SCAP	SREBP cleavage-activating protein
SCFAs	short-chain fatty acids
SFAs	saturated fatty acids
SFRP1	secreted frizzled-related protein 1
SG-GMSCs	supracrestal gingiva-derived mesenchymal stem cells
SHIP-TREND	Study of Health in Pomerania–TREND cohort
SIRT1	sirtuin 1
SMADs (Smad1/2/3/4)	SMAD family signaling proteins
SPM/SPMs	specialized pro-resolving mediator(s)
SRE	sterol regulatory element
SREBP/SREBP1	sterol regulatory element-binding protein / protein 1
STAT	signal transducer and activator of transcription
TAB1	TGF-β-activated kinase 1-binding protein 1
TAK1	transforming growth factor beta-activated kinase 1
TGF-β/TGF-β1	transforming growth factor beta / beta 1
TG	triglyceride
THP-1	human monocytic leukemia cell line THP-1
TLR2/TLR4	Toll-like receptor 2 / 4
TNF-α	tumor necrosis factor alpha
TNFR	tumor necrosis factor receptor
TRAF2/TRAF6	TNF receptor-associated factor 2 / 6
TRAP	tartrate-resistant acid phosphatase
VEGF	vascular endothelial growth factor
WBC	white blood cell
Wnt (Wnt1/3a/10b)	Wingless-related integration site family signaling proteins
